# Mixed muco-cutaneous pemphigoid: Clinical and immunological features of 15 cases

**DOI:** 10.3389/fimmu.2023.1134720

**Published:** 2023-03-15

**Authors:** Raphaël Janela, Norito Ishii, Marion Castel, Fabienne Jouen, Lucie Cellier, Philippe Courville, Pascal Joly, Vivien Hébert

**Affiliations:** ^1^ Department of Dermatology, Rouen University Hospital, Normandie University, Rouen, France; ^2^ Department of Dermatology, Kurume University School of Medicine, Kurume, Japan; ^3^ INSERM Unit U1234, PANTHER, Normandie University, Rouen, France; ^4^ Department of Anatomopathology, Rouen University Hospital, Rouen, France

**Keywords:** bullous pemphigoid, mucous membrane pemphigoid, new entity, immunoblot (western blot), direct electro immunomicroscopy

## Abstract

**Introduction:**

We describe a series of patients whose auto-immune bullous skin disease (AIBD) of the dermal-epidermal junction (DEJ) was characterized by clinical, immunological and ultrastructural features intermediate between bullous pemphigoid (BP) and mucous membrane pemphigoid (MMP), and a recalcitrant course.

**Patients and Methods:**

From the database of the French reference centre for AIBD, we screened all the patients who were referred for an AIBD of the DEJ with a mucosal involvement, who neither met the diagnostic criteria for the diagnosis of BP, nor were typical of MMP. Sera were analysed by NC16A-ELISA and immunobloting against the C-terminal and LAD-1 parts of BP180. Skin biopsies were studied by direct immunoelectron microscopy (IEM).

**Results:**

Fifteen patients (4 males, 11 females) of mean age 70.8 ± 11.8 years were included. The mucosal involvement was localized in oral cavity in all cases and in pharyngeal/laryngeal or genital area in 8 (53%), and 6 patients (40%), respectively. No patient had ocular involvement, nor atrophic or fibrosing scars. All patients had extensive skin lesions (mean BPDAI score =65.9 ± 24.4), which predominated on the upper body part. Direct IEM performed on 8 patients showed IgG deposits on the lamina lucida in all cases, and the lamina densa in 5 cases. All sera recognized NC16A, while none recognized BP-230 in ELISA. 10 out of the 13 tested sera (76.9%) contained IgG which recognized the C-terminal domain of BP180 and 10 sera (76.9%) the LAD-1 domain of BP180. Patients poorly responded to super potent topical corticosteroids and were treated with oral corticosteroids ± immunosuppressant in 13 cases (86.6%).

**Conclusion:**

This mixed muco-cutaneous pemphigoid differs from BP by the younger age of patients, multiple mucosae involvement, circulating antibodies against both the C- and N-terminal part of BP180, and very poor response to topical CS. It differs from MMP by extensive inflammatory skin lesions, absence of ocular involvement and atrophic/fibrosing scars.

## Highlights

- Some patients with junctional autoimmune bullous disease have clinical atypical involvements as well as serological profile that overlap between bullous pemphigoid (BP) and mucous membrane pemphigoid (MMP).
- We described patients who differ from BP by the younger age of patients, deep mucosae involvement but from MMP by extensive inflammatory skin lesions, absence of ocular involvement and atrophic/fibrosing scars. Histologically and serologically, patients had findings that could observed both in BP and MMP.

## Introduction

Bullous pemphigoid (BP) and Membrane Mucous Pemphigoid (MMP) are the two most common autoimmune bullous skin diseases (AIBD) of the dermal epidermal junction (DEJ).

BP classically occurs in the elderly and is characterized by a severe pruritic blistering eruption. BP is associated with autoantibodies targeting the NC16-A domain of BP180 and BP230, which are two components of the hemidesmosomes (1–7). Clinical criteria have been proposed in 1998 to differentiate BP from other AIBD (5), that have been re-evaluated in 2004 using immunoblot analysis (8). The presence of at least 3 of these 4 criteria: no mucosal lesion, no predominant damage to the head and neck, age older than 70 years, no atrophic scar; allows to predict the diagnosis of BP with a 86% sensitivity and a 90% specificity (5).

One the other hand, MMP describes a group of chronic AIBD of the chorio-epithelial or DEJ. Typical presentation is characterized by predominant or exclusive mucosal lesions which can involve oral, naso-pharyngeal, laryngo-tracheal, genital, esophageal, anal and ocular mucous membranes (9–11). Skin lesions are often limited and evolve to atrophic scars. MMP is mediated by autoantibodies directed against various antigens of the basement membrane zone (BMZ) including BP180, laminin 332, and type 7 collagen (12).

Numerous atypical forms of BP have been described, which account for around 20% of BP patients (13–15). A few atypical forms of MMP with extensive skin lesions on inflammatory skin have also been reported (16, 17). We recently treated patients with clinical features intermediate between BP and MMP. These patients were younger than the usual age of patients with the classical type of BP, and had both oral and laryngeal/pharyngeal involvement, and extensive inflammatory skin lesions. They had a common histological and immunological profile characterized by a subepidermal blister with polynuclear eosinophils, linear IgG deposits on the DEJ by DIF and the presence of anti-BP180 Abs using the commercially available ELISA, which detects antibodies against the NC16-A region (18, 19). To assess whether the clinical and immunological features of these patients were anecdotal or corresponded to a rare subgroup of AIBD of the DEJ, we screened all the patients who were referred to our centre for an AIBD of the DEJ with mucosal involvement, who neither met the diagnostic criteria proposed by Vaillant et al. for the diagnosis of BP, nor were typical of MMP.

We found 15 patients with the same clinical phenotype, overlapping between BP and MMP. These patients whose mean age was more than 10 years younger than “classical” BP patients presented with extensive inflammatory skin lesions, which predominated on the upper part of the body in many of them, and were associated with almost constant oral and extra-oral mucosal involvement. However, none of them had ocular involvement, nor atrophic or fibrosing scars. These clinical features which were reminiscent of both BP and MMP, were associated with: i) the detection of circulating antibodies which recognized both the NC16A domain and the C-terminal part of BP180, with no anti-BP230 antibodies, ii) the *in vivo* localization of IgG deposits on both the lamina lucida and the lamina densa in half of the cases and iii) a refractory course with poor response to super potent topical corticosteroids. Based on these characteristics, we suggest that it is a new entity that overlaps between BP and MMP as a mixed muco-cutaneous pemphigoid (MMCP).

## Patients and methods

### Inclusion criteria

Inclusion criteria were: i) skin lesions suggestive of AIBD of the DEJ with large tense blisters associated with mucosal involvement, which however did not meet 3 of the 4 clinical criteria proposed for the diagnosis of BP (5, 8); ii) ii) a histological picture of subepidermal blister with linear IgG and/or C3 deposits on the DEJ by direct immunofluorescence (DIF), and iii) labelling of the epidermal side of the detachment by indirect examination of patients’ serum on NaCl-split skin. Exclusion criteria were: i) the presence of IgA deposits on the DEJ by DIF, ii) a dermal labelling by IIF on salt split skin, iii) the recognition of collagen 7 or laminin γ1 by ELISA and/or immunoblot on dermal extract.

### Clinical data

The following clinical and biological data were recorded at the time of diagnosis: gender, age, drug intake, number of daily new blisters (< or ≥ 10), disease severity as measured by the bullous pemphigoid disease area index (BPDAI) (20, 21), and blood eosinophil count.

During patients’ follow-up, treatment efficacy and safety outcomes were recorded: proportion of patients who achieved disease control at Day 21, and one-year complete remission, and one-year mortality rate.

### Serological assays

#### Anti-BP180 and anti-BP230 autoantibody detection

Anti-BP180 and anti-BP230 autoantibodies were detected in serum using specific enzyme-linked immunosorbent assays (ELISA EUROIMMUN^®^) in the Immunology laboratory of the University Hospital of Rouen. No further dilution was performed when titres were ≥ 200 U/mL.

#### Detection of antibodies directed against non-NC16A parts of BP-180

Patient’s sera were also analysed by Dr Ishii from the Kurume University Hospital of Medicine, Japan using immunobloting on recombinant proteins directed against the LAD-1 (524-1497aa of human BP180) and the C-terminal (1193-1497aa of human BP180) part of BP180 in order to detect autoantibodies targeting epitopes other than NC16A. Out of the 13 patients, 11 sera were analysed, since sera from two patients were not available.

### Histological analysis and direct immunoelectron microscopy

All examinations have been performed in the pathology laboratory of the Rouen University Hospital. Histological analysis by hematoxylin-eosin staining was performed in all patients. Direct immunoelectron microscopy examination of skin biopsies from 8 patients. Skin biopsies were collected in a Hanks’ balanced salt solution. Thereafter, fragments were incubated with anti-IgG, peroxidase-coupled antibodies. After fixation in Karnovsky’s fixative, revelation in DAB and post-fixation in osmium, the samples were included in epoxy resin. Semi-thin (± ultrathin) sections were made to demonstrate immune-staining by electron microscopy (22).

### Statistical analysis

Statistical analyses were performed using GraphPad Prism version 9.0 (San Diego, CA, USA).

## Results

### Patients’ characteristics

#### Clinical characteristics of patients

From January 2010 to December 2020, 233 patients were referred to our centre for an AIBD of the DEJ. Of them, 15 patients (6.4%) corresponded to the inclusion criteria and were analysed.

**Figure 1:**
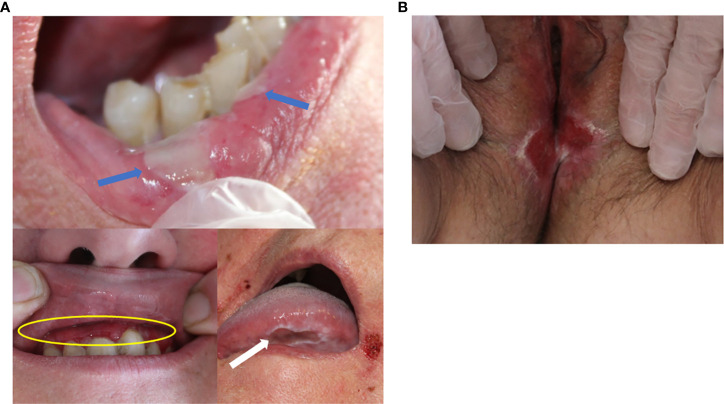
**(A)** Oral mucosal lesions. Blue arrows: labial blisters. Yellow circle: erosive gingivitis. White arrow: tongue ulceration. **(B)** Genital mucosal lesions. Erosive vulvitis.

**Figure 2:**
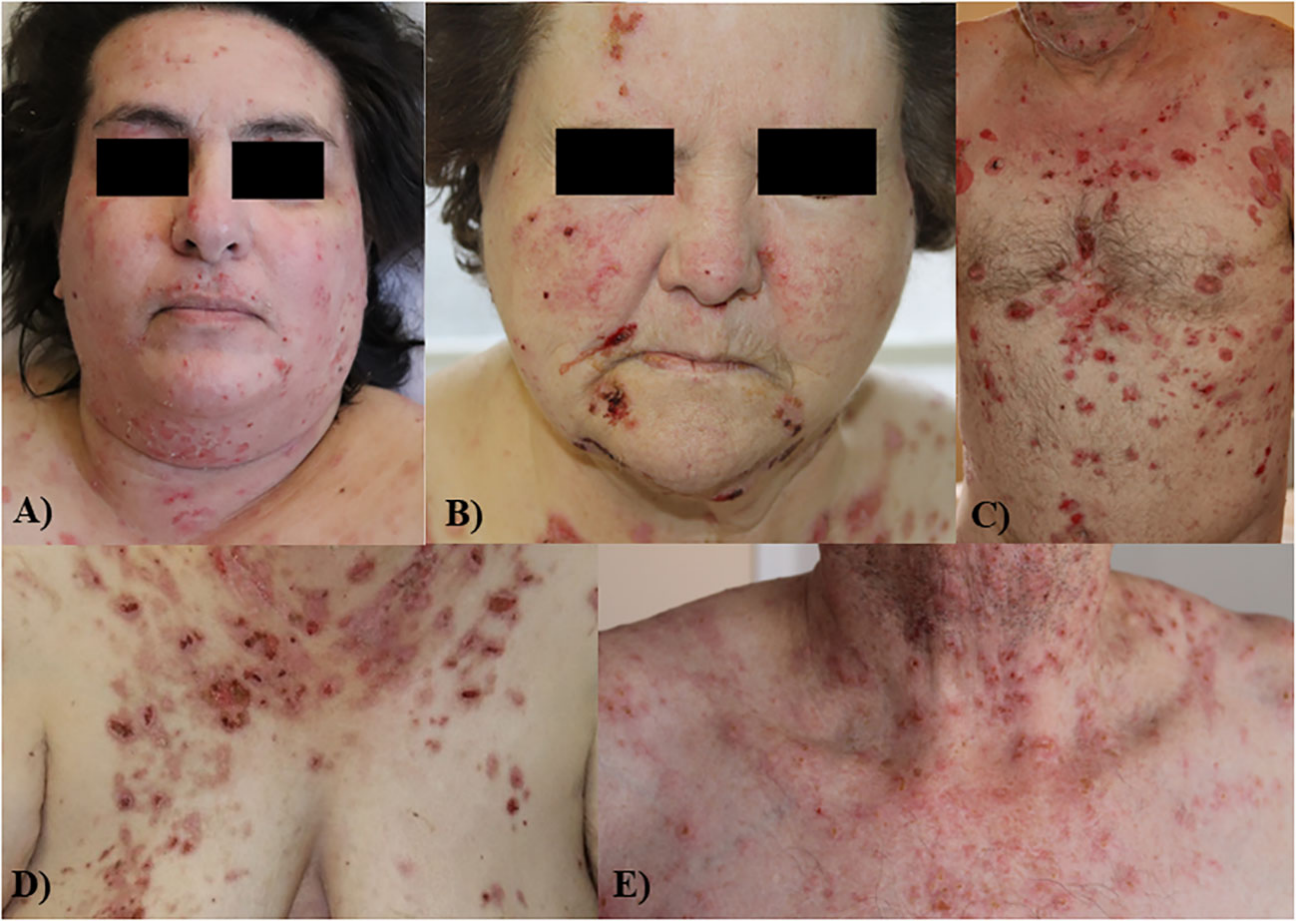
Patients with unsual severe involvement of the face **(A, B)**, upper part of the trunk **(C)** and neck **(D, E)**.

Clinical characteristics of the patients are described in **Table 1**. The male/female ratio was 0.36, and mean age 70.8 ± 11.8 years. Eight of the 15 patients were < 70 years old. All patients had oral mucosal involvement (**Figure 1A**) and 12 of 15 had an extra-oral involvement: 8 had a deep pharyngeal or laryngeal involvement (53%), and 6 had genital involvement (40%) (**Figure 1B**). No patient had ocular involvement. The mean BPDAI mucosal subscore was 13.3 ± 7. 5. Fourteen patients (93.3%) had an extensive skin involvement at the time of diagnosis, with a mean BPDAI total activity score of 65.9 ± 29.4, and a mean skin subscore of 52.6 ± 21.2. In particular, the blisters/erosions subscore was particularly high: 35± 12.5 out of 120. Ten patients (66.6%) had severe skin lesions on the head, neck and/or upper trunk (**Figure 2**). None of the patients had cicatricial or scarring skin or mucosal lesions. Surprisingly, only three patients suffered from neurological disorders.

**Table 1:** Patients characteristics.

*1. Clinical characteristics *	
**Number of patients (*n*)**	15
**Sex, n (%)**	
Male	4 (26.7)
Female	11 (73.3)
**Age, years**	
Mean age ± SD	70.8 ± 11.8
**Number of daily new blisters, n (%)**	
< 10 blisters per day	1 (6.7)
≥ 10 blisters per day	14 (93.3)
**BPDAI, mean ± SD**	
BPDAI total	65.9 ± 29.4
Activity of total skin involvement	52.6 ± 21.2
Blisters/erosions	35 ± 12.5
Erythema/urticaria	17.6 ± 10.7
Activity of mucosal involvement	13.3 ± 7.5

*2. Immunobiological characteristics *	
**Serum anti-BP180 NC16A ELISA**	
Number of patients (*n*)	13
Positive	13
Number of patients with titer ≥ 200, n (%)	10 (76.9)
**Eosinophilia value**	
Number of patients (*n*)	13
Mean ± SD (G/L)	1 ± 0.9
≥ 0,7 G/L, n (%)	6 (46.2)
**Indirect immunofluorescence**	
Number of patients (*n*)	13
Epidermic positivity, n (%)	11 (84.6)
Dermic positivity, n (%)	0 (0)
**Direct Immunoelectron Microscopy**	
Number of patients (*n*)	8
Lamina lucida IgG deposits	8
Lamina densa sIgG deposits	6

*3. Treatments efficacy *	
**Number of patients (*n*)**	15
**Control of disease at day 21 with topical CS alone**	1 (6.6)
**Use of at least one systemic treatment, n (%)**	13 (86.6)
**Mean number of lines of treatment ± SD**	2.4 ± 1.2
**Different treatment used, n (%)**	
Oral corticosteroids	13 (46.2)
Cyclins	6 (46.2)
Methotrexate	5 (38.5)
Mycophénolate mofetil	4 (30.8)
Dapsone	2 (15.4)
Rituximab	2 (15.4)
Omalizumab	2 (15.4)

Abbreviations: SD, standard deviation; BPDAI, bullous pemphigoid disease area index; BP, bullous pemphigoid.

#### Biological, histological and immunological findings

Blood eosinophilia (>0.5G/L) was present in 6 patients. According to the inclusion and exclusion criteria, all sera showed a labelling of the epidermal side of the detachment by IIF on salt-split skin, while no serum labelled the dermal side. All sera contained anti-BP180 antibodies without anti- BP230 antibodies by ELISA. Ten patients (76.9%) had anti-BP180 antibody ELISA values higher than 200 U/mL, which corresponds to the highest value detected by the kit.

Thirteen sera were analysed by immunoblot. All sera contained IgG which recognized the recombinant protein corresponding to the NC16A domain. In addition, 10 sera (76.9%) contained IgG which recognized the C-terminal domain of BP180, and 10 sera (76.9%) had IgG against the LAD-1 domain of BP180.

Histological analysis performed in all patients didn’t evidence a particular pattern. All patients had a typical subepidermal blister with polynuclear eosinophils. Polynuclear neutrophils were observed in 1 patient and lymphocytes infiltrate in 1 other patient.

**Figure 3:**
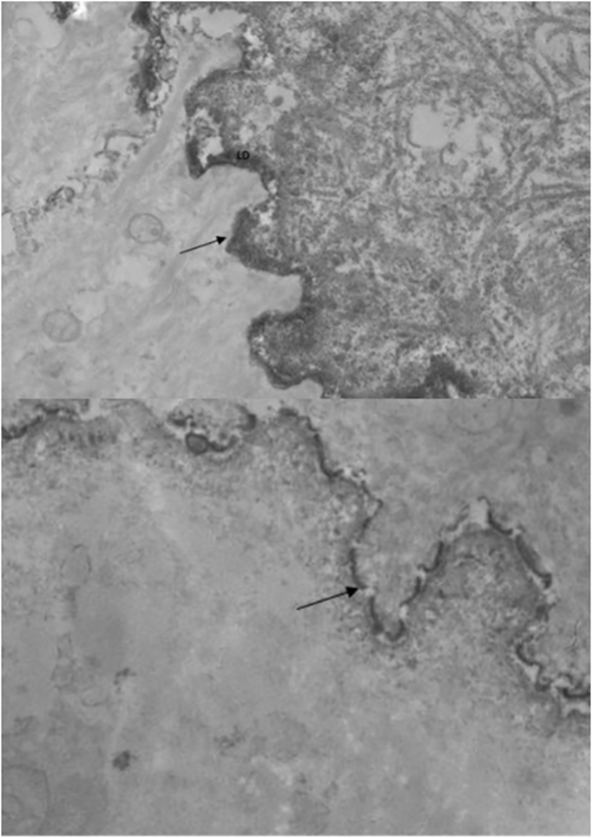
Electro immunomicroscopy IgG deposits on both lamina densa and lamina lucida in two patients.

Skin biopsies from 8 patients were examined by direct immunoelectron microscopy. All eight patients had IgG deposits in the lamina lucida, and 5 patients also had a labelling of the lamina densa (**Figure 3**). No deposits were observed within the hemidesmosomes.

#### Patients’ course and treatment efficacy

All patients were initially treated with super potent topical CS. Disease control was achieved by Day 21 in only one patient (6.6%) and was further achieved during the evolution in only 2 cases with topical CS alone. Thus, 13 patients (86.6%) needed a second-line treatment which corresponded to oral prednisone in all cases, either alone (n=3) or combined with an immunosuppressant (methotrexate, mycophenolate mofetil, rituximab) and/or immunomodulator (dapsone, cyclins, omalizumab) in 10 cases. The mean number of treatments lines was 2.4 ± 1.2. Despite the use of a second- or third-line treatment, only 6 out of the 15 patients (40%) achieved a complete remission after 12 months of follow-up.

## Discussion

We report a series of 15 patients presenting an atypical form of AIBD of the DEJ characterized by clinical, immunological and ultrastructural features intermediate between BP and MMP **Table 2**. These patients were characterized clinically by a rather young age, multiple oral and extra-oral mucosal involvement, extensive skin lesions, and a recalcitrant course. Immunological analysis of these sera showed the presence of anti-BP180 antibodies without anti-BP230 antibodies in all cases. Interestingly, anti-BP180 antibodies were not only directed against the NC16A domain, but also against the COOH-terminal domain of BP 180. Accordingly, *in vivo* IgG deposits were localised in the lamina lucida and in the lamina densa in 5 of the 8 biopsies examined by direct IEM.

**Table 2:** Characteristics of patients arguing against BP or MMP.

Against BP	Against MMP
Young age	Extensive cutaneous involvement
Head and neck involvement	Absence of opthalmic involvement
Deep mucosal involvement	Absence of scarring lesions
ELISA: absence of anti-BP230 Abs	ELISA: constant positivity of anti-BP180 Abs
Immunoblot: auto-Ab targeting the COOH-term domain of anti-BP180	
Poor response to conventionnal treatments	

Despite the fact that mucosal involvement has been reported in 20% of BP patients (14, 15, 23), the clinical phenotype of the patients reported in the present study differed from the main characteristics of BP patients including i) a mean age more than 10-year younger (70.8 ± 11.8 years versus around 83 years in French series (24, 25), including half of the patients who were < 70 years old; ii) the frequent involvement of mucosae other than the oral cavity, since a deep pharyngeal or laryngeal involvement was observed in half of these patients, while it is rarely observed in patients with BP, and iii) the predominance of skin lesion on the head, neck and upper trunk in 66.6% of patients, which is reminiscent of MMP rather than BP. Moreover, only 3 out of the 15 patients had associated debilitating neurological disorders, as it has been classically reported in BP patients (26). Finally the serological profile of these patients, which was characterised by exclusive anti-BP180 antibodies with no anti-BP230 antibodies, differed from that of classical BP sera since only 26% of BP sera studied by ELISA in a large serological study had this BP180 positive/BP230 negative immunological profile (27).

The involvement of both the oral and extra oral mucosa, the predominance of skin lesion on the head and neck and upper part of the trunk in half of the patients, as well as the presence of circulating antibodies directed against BP180 with no anti-BP230 antibodies might suggest the diagnosis of MMP. However, all these patients had extensive inflammatory skin lesions with very high mean BPDAI skin activity sub-score of 52, which is extremely unusual in MMP. Interestingly, both the blister/erosion and the erythema/urticaria skin sub-scores were elevated, indicating the inflammatory character of skin lesions, which is not characteristic of MMP. Additionally, none of the patients had fibrosing nor atrophic scars, and none of them develop ocular involvement, which strongly argued against the diagnosis of MMP.

In accordance with the patients’ clinical features which overlapped between BP and MMP, immunoblot analysis of the patients’ sera using recombinant proteins corresponding to the C- and N-terminal parts of BP180 showed that 76.9% of these sera contained IgG antibodies directed against the LAD-1 and COOH-terminal parts of BP180, in addition to NC16A. These results are in accordance with the findings by Hoffman et al. who reported that 56% of sera from BP patients with mucosal involvement contained antibodies directed against the C-terminal domain of BP180, which confirms that this part of the protein seems implicated in the mucosal involvement of AIBDs of the DEJ, as observed in MMP (28). Indeed, some studies suggested that the presence of mucosal lesions in BP correlated with levels of IgG against the COOH-terminal epitope of BP180 that is located deeper than NC16A (19). Interestingly, whereas direct IEM examination of patients’ skin showed *in vivo* antibody deposits on the lamina lucida in all the 8 biopsies examined, 5 patients whose serum contained antibodies directed against the C-terminal domain of BP180 also had *in vivo* IgG deposits on the lamina densa, in addition to the lamina lucida.

Finally, this particular clinical and immunological phenotype seems associated with a recalcitrant course since only 2 patients achieved disease control on topical CS alone whereas it usually allows it in 75% to 98% of cases (24, 25, 29, 30). The 13 others patients were secondarily treated with oral CS alone or combined with immunosuppressants.

Overall, while the overlapping distribution of antibodies directed against the C- and N-terminal part of BP80 has been described for many years in BP and MMP patients, we describe a corresponding particular clinical phenotype which is characterized by clinical, immunological and ultrastructural features intermediate between BP and MMP occurring in younger patients and associated with a recalcitrant course.

## Data availability statement

The raw data supporting the conclusions of this article will be made available by the authors, without undue reservation.

## Ethics statement

Ethical review and approval was not required for the study on human participants in accordance with the local legislation and institutional requirements. The patients/participants provided their written informed consent to participate in this study. Written informed consent was obtained from the individual(s) for the publication of any potentially identifiable images or data included in this article.

## Author contributions

RJ: acquisition of data, drafting the manuscript NI: acquisition of data MC: acquisition of data FJ: acquisition of data LC: analysis of data PC: analysis of data PJ: design, draft and review of the manuscript VH: design, draft and review of the manuscript. All authors contributed to the article and approved the submitted version.
